# Supplementary material for: The potential impact of advanced footwear technology on the recent evolution of elite sprint performances
Source: PeerJ. 2023 Nov 27;11:e16433. doi: 10.7717/peerj.16433 (PMC10688325; doi:10.7717/peerj.16433)
Supplement: Supplemental Information 3 [file peerj-11-16433-s003.pdf]

Supplementary File 3: P-values of the Wilcoxon-Mann-Whitney tests assessing the null hypothesis that it is equally likely that a value chosen at random from one year is greater or less than a value chosen at random from another year's population.

Top 100

Table 1 Men's 100m

	2016	2017	2018	2019	2021
2017	1				
2018	1	0.638283			
2019	1	1	1		
2021	1	0.309905	1	1	
2022	1	0.00015	0.00196	0.003115	0.062573

Table 2 Men's 110m hurdles

	2016	2017	2018	2019	2021
2017	1				
2018	1	1			
2019	1	1	0,714507		
2021	0,097206	0,025	0,000356	0,003474	
2022	0,003464	0,000552	4,56E-06	3,78E-05	0,726513

Table 3 Men's 200m

	2016	2017	2018	2019	2021
2017	1				
2018	1	1			
2019	1	1	1		
2021	1	1	1	1	
2022	0,052459	0,001119	0,046565	0,014442	0,047502

Table 4 Men's 400m

	2016	2017	2018	2019	2021
2017	1				
2018	0,572175	1			
2019	1	1	1		
2021	1	1	1	1	
2022	0,052919	0,627806	1	0,078112	0,272402

Table 5 Men's 400m hurdles

	2016	2017	2018	2019	2021
2017	1				
2018	1	1			
2019	1	1	1		
2021	1	1	1	1	
2022	0,972897	1	1	0,388271	0,973264

Table 6 Women's 100m

	2016	2017	2018	2019	2021
2017	1				
2018	1	0,466331			
2019	1	1	1		
2021	0,03227	0,02574	0,139897	0,011156	
2022	4,53E-07	4,06E-06	2,3E-06	4,28E-08	0,003582

Table 7 Women's 100m hurdles

	2016	2017	2018	2019	2021
2017	1				
2018	1	1			
2019	1	1	1		
2021	1	0,004164	0,065499	0,129516	
2022	0,746016	0,001377	0,023704	0,042453	1

Table 8 Women's 200m

	2016	2017	2018	2019	2021
2017	1				
2018	1	0,492091			
2019	1	1	1		
2021	1	0,265376	1	0,002601	
2022	0,085241	0,000304	0,043264	2,27E-06	0,265376

Table 9 Women's 400m

	2016	2017	2018	2019	2021
2017	1				
2018	0,804407	0,371019			
2019	1	0,702147	1		
2021	1,35E-05	2,2E-07	0,001172	6,98E-05	
2022	4,93E-05	5,75E-07	0,002364	0,000161	1

Table 10 Women's 400m hurdles

	2016	2017	2018	2019	2021
2017	1				
2018	1	1			
2019	1	1	1		
2021	1	1	0,346209	0,375669	
2022	1	0,608207	0,098102	0,080571	1

Top 20

Table 11 Men's 100m

	2016	2017	2018	2019	2021
2017	1				
2018	1	0,800315			
2019	1	1	1		
2021	0,972321	0,017573	0,297023	0,059996	
2022	1	0,021544	0,33573	0,078011	1

Table 112 Men's 110m hurdles

	2016	2017	2018	2019	2021
2017	1				
2018	1	1			
2019	1	1	1		
2021	0,303898	0,57473	0,003671	0,082601	
2022	0,109341	0,290332	0,003215	0,062877	1

Table 13 Men's 200m

	2016	2017	2018	2019	2021
2017	1				
2018	1	0,062617			
2019	1	0,175687	1		
2021	1	0,685787	1	1	
2022	0,175687	0,000813	0,232567	0,269417	0,154942

Table 14 Men's 400m

	2016	2017	2018	2019	2021
2017	1				
2018	1	1			
2019	1	1	1		
2021	1	1	1	1	
2022	1	1	1	1	1

Table 15 Men's 400m hurdles

	2016	2017	2018	2019	2021
2017	1				
2018	1	1			
2019	1	1	1		
2021	0,407642	0,524398	0,53255	0,160764	
2022	0,197812	0,338821	0,407642	0,160764	1

Table 16 Women's 100m

	2016	2017	2018	2019	2021
2017	1				
2018	1	1			
2019	1	1	1		
2021	1	0,517847	0,006641	0,012547	
2022	0,317904	0,018162	4,46E-05	0,004229	0,494987

Table 17 Women's 100m hurdles

	2016	2017	2018	2019	2021
2017	0,711735				
2018	1	1			
2019	0,711735	1	0,699526		
2021	0,045484	0,231794	0,114568	0,614191	
2022	0,001485	0,001485	0,005646	0,014589	0,076741

Table 18 Women's 200m

	2016	2017	2018	2019	2021
2017	1				
2018	1	1			
2019	1	1	1		
2021	0,012536	0,016489	0,003203	0,007309	
2022	0,007309	0,007309	0,001137	0,005665	1

Table 19 Women's 400m

	2016	2017	2018	2019	2021
2017	1				
2018	1	1			
2019	1	1	1		
2021	0,026829	0,011861	0,054195	0,010109	
2022	0,467379	0,615339	0,757428	0,054195	1

Table 20 Women's 400m hurdles

	2016	2017	2018	2019	2021
2017	1				
2018	1	1			
2019	1	1	1		
2021	1	1	0,074017	0,574967	
2022	0,103321	0,574967	0,005963	0,074017	1
