# Supplementary material for: The potential impact of advanced footwear technology on the recent evolution of elite sprint performances
Source: PeerJ. 2023 Nov 27;11:e16433. doi: 10.7717/peerj.16433 (PMC10688325; doi:10.7717/peerj.16433)
Supplement: Supplemental Information 4 [file peerj-11-16433-s004.pdf]

Supplementary File 4: list of shoes used in sprint events during the 2021-2022 seasons considered to include AFT

AFT was defined as per Healey et al. (2022), whereby a superspike incorporates "a combination of lightweight, compliant and resilient foams (and/or air pods) with a stiff (nylon, PEBA, carbon-fiber) plate".

- Adidas Adizero Prime SP2
- Adidas Adizero Avanti TYO
- Asics Metaspeed SP 0
- New Balance FuelCell Sigma SD-X
- New Balance FuelCell SuperComp PWR-X
- New Balance SuperComp MDX
- Nike Air Zoom Maxfly
- Nike Air Zoom Victory
- Puma evoSPEED Tokyo Nitro
- Puma evoSPEED Tokyo Nitro 400
